# Recent Challenges in Insulin Delivery Systems: A Review

**DOI:** 10.4103/0250-474X.42968

**Published:** 2008

**Authors:** M. M. Al-Tabakha, A. I. Arida

**Affiliations:** Department of Pharmaceutics, Faculty of Pharmacy and Health Sciences, Ajman University of Science and Technology Network, P.O. Box 2202, Al-Fujairah, UAE; 1 Faculty of Pharmacy, Philadelphia University, P.O.Box 1, Postal Code 19392, Jordan

**Keywords:** Diabetes mellitus, non-invasive delivery systems, insulin, Exubera^®^

## Abstract

Relatively, a large percentage of world population is affected by diabetes mellitus, out of which approximately 5-10% with type 1 diabetes while the remaining 90% with type 2. Insulin administration is essential for type 1 patients while it is required at later stage by the patients of type 2. Current insulin delivery systems are available as transdermal injections which may be considered as invasive. Several non-invasive approaches for insulin delivery are being pursued by pharmaceutical companies to reduce the pain, and hypoglycemic incidences associated with injections in order to improve patient compliance. While any new insulin delivery system requires health authorities' approval, to provide long term safety profile and insuring patients' acceptance. The inhalation delivery system Exubera^®^ has already become clinically available in the United States and Europe for patients with diabetes as non-invasive delivery system.

Insulin is a hormone secreted from the β cells of the islets of Langerhans, specific groups of cells in the pancreas. Insulin is a protein consisting of two polypeptide chains, one of 21 amino acid residues and the other of 30, joined by two disulfide bridges. It was isolated in 1921 with its first clinical use in 1922^1^. Nowadays, the major advances achieved in this area include the human insulin analogue synthesis from the recombinant DNA techniques^2^,^3^.

Insulin is a key player in the control of intermediary metabolism^4^. It has profound effects on both carbohydrate and lipid metabolism and has significant influence on protein and mineral metabolism^5^. Consequently, abnormal insulin level or responses have widespread and devastating effects on many organs and tissues^6^,^7^.

Inadequate lowering of blood glucose level to normal level results in diabetes. Normal people have fasting sugar level less than 110 mg/dl as set by the American Diabetes Association. Diabetes is either type 1, as a result of frank deficiency of insulin or type 2, due to resistance of tissue to respond insulin. The former onset happens in the childhood while the latter in adulthood. In type 1 diabetic patients, the treatment is based on administering insulin and diet control. For type 2 diabetic patients, although insulin is not required initially, but may require the administration of insulin because of decrease in the insulin secretion^8^,^9^. These accounts for 7% of type 2 diabetic patients.

Diabetes affects a large percentage of population around the world and has assumed epidemic dimensions^10^,^11^. The current estimate of the number of diabetic patients in the world is 171.2 million (2.8%) in the year 2000 and predicted to be 366.2 million (4.4%) by the year 2030^12^. In countries like Jordan, UAE and Saudi Arabia, this has reached to the epidemic proportion of > 9% of the total population. The percentage will be much higher considering the adult population, since type II diabetes is mainly an adulthood disease^13^. Type II diabetes is the main manifestation of diabetes mellitus^14^. The percentage of type I diabetic patients in the total diabetic patients is 5-10%^15^. This means that in the year 2000, there were about 12.8 million of types I diabetic patients depending on insulin administration. In addition, 7% of the remaining 158.4 million i.e. 11.1 million also require the administration of insulin making a total of 23.9 million insulin dependants. Diabetes, if not controlled, can result in complications to virtually every system of the body. Some of the serious complications of diabetes include blindness (due to diabetic retinopathy), end-stage renal disease (due to diabetic nephropathy), lower extremity amputations (resulting from diabetic neuropathy), decreased ability to fight infection and impotence or sexual dysfunction^16^. Diabetes mellitus (DM) is a major risk factor for heart disease, stroke and peripheral vascular diseases.

The patients requiring insulin may have to take more than 60,000 injections throughout their life^17^. In type 1 diabetes, good glycemic control usually requires at least two or more often three or more daily insulin injections. Such invasive and intensive technique urges the search for alternative more pleasant methods for administering insulin^18^. It is predicted that as soon as non-invasive insulin delivery technology becomes available, 2.5% of the total diabetic population will use it with projected rapid increase by time.

The traditional and most predictable method for the administration of insulin is by subcutaneous injections. This method is often painful and hence, deterrent to patient compliance especially for those requiring multiple dose injections of four times a day. Also, there have been reports of hypoglycemic episodes following multi dose injections of insulin^19^. Several new approaches to the method have been adopted to decrease the suffering of the diabetic patients including the use of supersonic injector, infusion pump, sharp needles and pens. While some of them eased the pain encountered by the diabetic patients, they offer incomplete convenience. Even though the ultimate goal would be to eliminate the need to deliver insulin exogenously and regaining the ability of patients to produce and use own insulin, new concepts are currently explored to deliver insulin using oral, pulmonary, nasal, ocular and rectal routes. The success of the route of administration is judged on the basis of its ability to elicit effective and predictable lowering of blood glucose level and therefore minimizing the risk of diabetic complications. It is clear that several difficulties have to overcome with the use of formulation and application devices technology. The various explored routes are reviewed in this review paper.

## CURRENT INSULIN DELIVERY SYSTEMS

Insulin delivery systems that are currently available for the administration of insulin include syringes, insulin infusion pumps, jet injectors and pens. A concise review of these delivery devices has been described as under:

### Insulin syringes:

Insulin syringes that were introduced initially were large and heavy with reusable glass plungers and barrels with a long, large bore needle. These syringes underwent significant changes over the years. Today, many insulin injection syringes are available in the marketplaces that are derived from plastics being light in weight, disposable and versatile in use of variety of microfine needles^20^. These syringes increase patient comfort and offer convenience, thus better patient compliance^21^.

Insulin syringes are characterized by three factors, i.e. needle gauge, needle length and syringe capacity. The manufacturers of the syringes offer a wide array of sizes and styles. Almost all syringes available today are disposable and come with microfine needles^22^. The proper selection of an appropriate syringe is based on many considerations, like chemical composition of the material from which syringes are made, syringe capacity^23^, ease with which air bubbles are removed, clarity of the markings on the syringe barrel and convenience of syringe disposal. In addition, one must consider the condition of the patient with respect to his/her ability to operate the syringe safely and effectively.

While conventional syringes offer a wide choice of products that are easy to read and operate, the disadvantages include their bulky construction and the requirement of time and practice to learn optimal syringe technique. Furthermore, the required syringe manipulations in the social setting (such as workplace, classroom and public places including department stores, playgrounds and restaurants) may be considered as drawbacks. In some cases, patients need to mix different types of insulin preparations in one syringe to meet their individual needs, which can be cumbersome and complicated. In patients with less dexterity, this may result in inaccurate doses, compromising their glycemic control.

### Insulin infusion pumps:

Continuous subcutaneous insulin infusion (CSII) is a way to simulate the physiology of daily insulin secretion^24^. The first CSII pump was introduced in the market in 1974^25^. By design, an insulin pump typically consists of a reservoir filled with insulin (e.g., Velosulin^®^BR), a small battery operated pump and a computer chip that allows the patient to control the insulin delivery. The pump is designed to provide a continuous supply of insulin infusion around the clock and can be adjusted as per the specific needs of the patient. Appropriate amounts of insulin are delivered into the body by the pump through a thin plastic tube known as an infusion set. Most of the factors that affect the variability of subcutaneous injections such as depth of injection and change of injection sites are avoided with pump systems. In these pumps, the insulin reservoir is connected to a subcutaneous catheter, which is changed every two to three days. Thus, advantageous for people who do not like injections as it is only necessary to insert a needle once every three to four days. Patient education by a healthcare team is a crucial component of successful insulin pump therapy.

Although the size of the initial designs was prohibitive, the technological advances in the pump designs of CSII led to the development of newer systems that are much smaller in size (approximately the size of a pager, i.e., 5×7.5 cm). These are relatively easier to operate than the earlier ones and can be carried conveniently in a shirt pocket. However, some patients may not like the idea of wearing a pump constantly or disconnecting the catheter before bathing or swimming.

Insulin pumps provide accuracy and greater flexibility in insulin delivery for patients according to their individual requirements, especially during travel. Some of the available infusion pumps have the ability to accurately deliver microdoses (0.1 units) of insulin. The newer devices are easy to use and carry and provide a small subcutaneous depot of unabsorbed insulin. The pump devices allow a patient to achieve a very tight control of plasma glucose levels and enhance the overall quality of life. However, if and when insulin delivery is interrupted by infusion set malfunction, needle displacement, pump dysfunction or lack of insulin in the reservoir, circulating insulin concentration drops rapidly causing problems^26^. This may be a great concern for some patients. However, patients who experience many hypoglycemic episodes may benefit from infusion pumps. When compared with optimized multiple daily insulin injections, CSII resulted in a modest but worthwhile improvement in blood sugar in adults with type 1 diabetes^27^.

The therapy with insulin pump is very expensive as compared to the use of traditional syringes and vials. In a clinical study, the cost/benefit ratio was found to be favorable only in those patients who were prone to frequent hypoglycemia. This is because of the lower hospitalization rate of such patient population. Also the health and management benefits of the use of the insulin pump outweighed that of multi dose injections as it provides more freedom, flexibility, and spontaneity in the person's daily life^28^.

### Insulin jet injectors:

Jet injectors, (introduced in the 1980s), are designed to deliver a fine stream of insulin transcutaneously at high speed and high pressure to penetrate the skin without a needle. The use of force on a fluid under considerable pressure through a very small opening allows such systems to deliver insulin without using a needle to pierce the skin. The dose is controlled by a dial-a-dose operation through a single component design in comparison to the conventional multicomponent syringe and vial method. The available jet injectors allow a dose range of two to 50 units of insulin and can deliver insulin in half-unit increments. Insulin that is administered by the jet injector method is absorbed rapidly without the risk of subcutaneous infection^29^. In gestational diabetes, jet injection therapy is associated with less antiinsulin antibody (AIA) production and better postprandial glycemia^30^.

The size and the cost of these jet injectors are considered unfavorably and often limit their routine use in patients with diabetes^31^. The potential for a decreased amount of absorbed insulin over repeated administration with jet injectors is a disadvantage. Additional concerns with jet injectors include pain or bruising at the administration site and the noise the injector makes upon delivery. Pressure may be difficult to adjust and the frequency of side effects seems to be significantly higher for young children^32^. However, jet injectors may be considered for patients suffering from needle phobia and for patients who suffer from severe lipomas^33^.

### Insulin pens:

Pen devices are novel in that they combine the insulin container and the syringe into a single modular unit. Insulin pens eliminate the inconvenience of carrying insulin and syringes. The first insulin pen (NovoPen^®^) was introduced by Novo Nordisk in 1987. Many pens are available since then in a variety of types and shapes. There are two main types of pens, one that is reusable and the other a prefilled device. In the former case, the patient must load an insulin cartridge prior to use. Regardless of the type, both pens hold cartridges containing from 1.5 ml to 3 ml of U100/ml insulin. The number of steps required to change an insulin cartridge with reusable pens varies between the different pen device manufacturers. Prefilled devices are well accepted in a bedtime insulin regimen for type 2 patients^34^.

Reusable insulin pens offer a wide range of advantages such as their durability, eliminating the need of cartridge refrigeration and flexibility in carrying three to five day supply. The pens also offer discreetness by resembling fountain pen. The refilled insulin pens are smaller in size and lighter in weight. They cause minimal pain due to the finest and shortest disposable insulin needles. In addition, they are quick and easy to use as they resemble the fountain pen; they are considered to be discreet.

In using insulin pens, the patient must attach a needle, prime the pen, set the dose by a dial and depress the plunger to administer the selected dose. There are several brands from which the patient with neuromuscular weakness and impaired manual coordination can select to employ the overall lowest force throughout delivery^35^. The manufacturers of the pen devices recommend keeping the needle separate and attaching only when ready to use. A study has shown that reusing insulin pen needles could help in reducing the economic burden of diabetes without leading to needle tip deformity and increased pain^36^. The needles for pens are available in varying lengths (from 8 mm to 12.7 mm) and varying gauges (from 29- to 31-gauge; the larger the gauge number, the smaller the diameter of the needle bore). The needles have a bevel on each end; one is intended to be inserted into skin and the other is to pierce the septum of the insulin cartridge.

The devices can add lifestyle flexibility and may result in better glycemic control^37^,^38^. The precision of insulin doses varies between different pens but remains better than that obtained in studies where traditional syringes were used^39^. Many newer generation pens are able to deliver 60 U at a time for type 2 patients. Insulin pens have become very popular in some countries such as France where over 50 percent of insulin-treated patients are using insulin pens^40^. Some studies indicated wider acceptability in elderly and adolescent patients with respect to easier and faster injection and greater comfort^41^.

While pens offer convenience, comfort, accuracy, discretion, durability and ease of storage^42^,^43^, patient education is essential in order to avoid operational errors, particularly when changing the cartridge in reusable pens. The proper use of insulin pens has been shown to enhance patient compliance during multiple injection regimen management.

## FUTURE TRENDS IN INSULIN DELIVERY SYSTEMS

Several companies are working on developing new ways of taking insulin, from pills to patches to mouth sprays to inhalers. Inhalers are furthest along in development and one has already been approved for marketing. Table 1 presents summary of the products under development as insulin delivery alternatives to injection.

**TABLE 1:** SUMMARY OF THE ALTERNATIVE INSULIN DELIVERY PRODUCTS BEING DEVELOPED

Name	Nature of the product	Company	Stage in the development
Exubera^®^	Inhaled insulin powder	Pfizer, Sanofi-Aventis and Nektar	FDA and EC approved (January, 2006)
AERx^®^ iDMS	Inhaled insulin solution	Aradigm and Novo Nordisk (Novo Nordisk bought all the developing rights)	Phase III in progress
Aerodose^®^	Inhaled insulin solution	Aerogen and Disetronic Medical Systems	Phase II completed
HIIP (Device Name Air^®^)	Inhaled insulin powder	Alkermes and Eli Lilly & Company	Clinical Trial-Phase III (for type 1 and 2)
Rapid Mist/Oralin	Mouth spray for buccal delivery (Rapid Mist is device, Oralin is insulin)	Generex Biotechnology	Completed phase II in Canada and Europe. Undisclosed in U.S.A. Oral-lyn™ (identical product) has been approved for commercial marketing and sale by the Ecuadorian Ministry of Public Health for the treatment of both Type-1 and Type-2 diabetes
EMISPHERE^®^ oral insulin tablets	Tablet	Emisphere Technologies	Phase II completed
NIN-058 (No trade name yet)	Tablet	Nobex Corporation and GlaxoSmithKline	Phase II in progress
Patch (No trade name yet)	Basal insulin patch	Altea Development Corporation	Phase I

AERx^®^ iDMS: AERx^®^ insulin diabetes management system. HIIP: Human insulin inhalation powder. FDA: Food and drug administration. EC: European commission.

Concise information on some of the prominent insulin delivery systems given as under:

### Insulin inhalers:

The lungs, on account of their large surface area^44^, are an ideal target for drug delivery and inhaled insulin (pulmonary insulin) represents one of the most promising alternatives to injection. The first attempt to deliver insulin by inhalation was made more than half century ago^45^. Clinical experience has shown that inhaled insulin has the potential to be an effective treatment in patients with diabetes, with particular utility in the treatment of postprandial hyperglycaemia^46^,^47^.

Several companies are working on insulin inhalers than any other insulin delivery option. Insulin inhalers would work much like asthma inhalers. The products fall into two main groups: the dry powder formulations and solution, which are delivered through different patented inhaler systems.

Exubera^®^, containing rapid-acting insulin in powder form, has been studied extensively in patients with type 1 and type 2 diabetes mellitus^48^. The AERx^®^ Insulin Diabetes Management System (AERx^®^ iDMS) delivers a liquid form of human insulin. Preliminary data indicate that patients converting from insulin injections to inhalation systems showed higher compliance to therapy, demonstrated by improved glycemic control. Other pulmonary insulin delivery systems, including ProMaxx^®^ (Epic Therapeutic- Baxter Healthcare Corporation), AIR^®^ (Alkermes, Eli Lilly), Spiros^®^ (Dura Pharmaceuticals and Eli Lilly), and Technosphere™-insulin Med Tone^®^ inhaler (Pharmaceutical Discovery Corp.), are also under investigation^49^. In humans, inhaled regular insulin is more rapidly absorbed than insulin from the subcutaneous injection. However, the efficiency of inhaled insulin is lower than that of subcutaneous injection because pulmonary delivery of insulin involves some loss of drug within the inhaler or mouth during inhalation.

Since, insulin is known to have growth-promoting properties; many clinicians are concerned about the possibility of long-term effects from the intraalveolar deposition of insulin within the lung. Safety data show that cough is the most common side effect. Pulmonary function tests have shown some changes in carbon monoxide diffusion in the lung, but further studies are needed to clarify the significance of these findings^50^. The long-term safety of these products has not been established. However, there is a strong need for studies on the basal insulin for prolonged pulmonary delivery as several companies are developing fast acting insulin for pulmonary delivery^51^,^52^.

The front-runner in the race for inhalable insulin appears to be Exubera^®^. The product is a joint-development program between Sanofi-Aventis and Pfizer, using a proprietary inhalation device and powdered insulin formulation developed by Nektar Therapeutics. Phase III trials had been completed, and the FDA has agreed to filling of New Drug Application (NDA) in March, 2005. In late January 2006, the FDA gave its approval for Exubera^®^ use in patients over 18 years of age for type 1 and type 2 diabetes. In Europe similarly, the product has been approved by the European Commission (EC) in January, 2006 for the treatment of type 1 and type 2 diabetes.

**Fig. 1:**
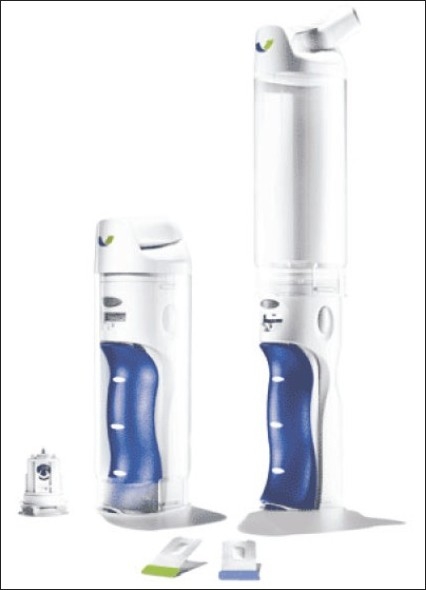
Exubera^®^ insulin inhaler with insulin blisters and the inhaler's insulin release unit (bottom left). The device is closed (left) for portability and extended (right) before use (Pfizer).

Exubera^®^ device (fig. 1) is about 25 cm long, has a base into which a packet of insulin powder is placed and a clear chamber above which the insulin powder is turned into an aerosol cloud for inhalation. The device delivers powdered insulin in 1 and 3 mg doses (approximately 3 and 9 units, respectively). Fine powder particles can stick together and become difficult to inhale effectively, reducing the chances of accurate dosing. Many inhalers (e.g. those used by some asthmatics) rely on a quick inhalation by the patient to try and force the particles apart. However, rather than rely on the ability of the patient to get their breathing right in order for effective treatment, Exubera^®^ provides its own energy to keep particles apart. The inhaler uses compressed air traveling at the speed of sound to create a cloud of insulin powder that the patient can then breathe in slowly and deeply to their lungs, where it dissolves into the blood stream. The inhaler requires no power source and the clear chamber ensures that the patient knows immediately when all the insulin has been inhaled.

**Fig. 2:**
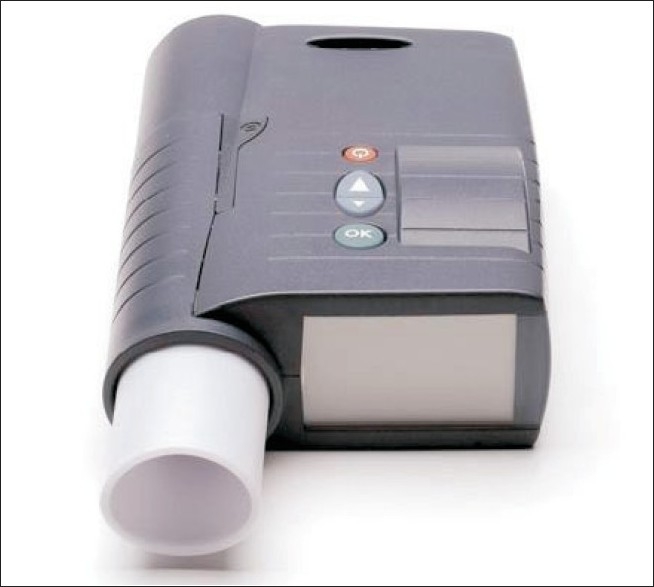
AERx^®^ insulin diabetes management system is an electromechanical device that delivers insulin from solution at correct rate and depth of breathing (Novo Nordisk). The device has capabilities of dosing increments by single-unit and data capture for dosing and compliance monitoring.

Novo Nordisk, a world leader in insulin manufacture and diabetes care, has bought all the manufacturing and development rights for AERx^®^ iDMS from Aradigm Corporation after ending early phase III trial^53^. This raises doubts about the success of inhalation delivery of insulin. The AERx^®^ insulin Diabetes Management System (AERx^®^ iDMS), shown in fig. 2 is 17.5 by 10 by 3.75 cm, produces a fine aerosol mist of liquid insulin that is efficiently deposited deep within the lung. The hand-held device visually guides the patient to breath at the correct rate and depth and automatically delivers insulin at the right point during inhalation, thus eliminating the effect of a poor breathing technique. The development of this ‘active breath control’ system in the AERx^®^ system makes the device exceptionally easy to use, ensuring that patients are able to consistently administer insulin. The patient can rest assured that “partial-dosing” is impossible. When the “green-light” is obtained, insulin will be delivered.

Clinical trials have shown that inhaled insulin delivered by the AERx^®^ iDMS controls blood glucose in the similar way as injected insulin in terms of absorption and onset of action like the rapid acting analogues, making it ideal for dosing immediately prior to a meal. This unique liquid formulation also allows tight control of glucose, since the dose may be increased just like injectable insulin in steps equivalent to 1 unit.

### Mouth sprays:

Mouth sprays deliver insulin through an aerosol spray and hence, they differ from inhalers. In mouth sprays, the insulin is absorbed through the inside of cheeks and in the back of mouth instead of lungs^54^,^55^. Two forms of mouth spray (Rapid Mist/Oralin) are being developed by Generex Biotechnology^56^,^57^. One of the forms is fast-acting whereas, another one covers the basal rate of insulin (the basal rate is the amount of insulin required throughout the day to keep blood sugars stable).

### Pills:

The concept of delivering insulin by mouth (“peroral” delivery) for absorption across the intestinal wall into the portal vein has long been regarded as a difficult challenge, but of substantial clinical and commercial potential. Presently, the biggest challenge with insulin pills is posed by the human digestive system. Either the gastrointestinal tract breaks the insulin down or the insulin passes out intact because it is unable to pass through the gastrointestinal membrane. Attempts have been made to overcome such obstacles. For example, insulin was complexed with cyclodextrins (CD) in order to improve its solubility and stability in the form of a dry powder, after encapsulation into poly (D,L-lactic-co-glycolic acid) (PLGA) microspheres^58^. Other examples include a delivery agent SNAC (sodium N-[8-(2-hydroxybenzoyl)amino] caprylate) that was incorporated with insulin^59^, a hyaluronan-insulin complex was prepared^60^ and calcium phosphate-PEG-insulin-casein (CAPIC) particles were produced for oral delivery of insulin^61^.

Several manufacturers are working on pills. In these pills, special molecules were attached to the insulin to help to reach its destination either by reducing insulin break down or, escorting the insulin through the gastrointestinal lining, or both. NOBEX Corporation has applied the technology that permits them to deliver insulin orally as an orally absorbed, bioactive conjugate. It has been shown to be safe, rapidly absorbed and demonstrated dose-dependent, glucose-lowering effects in animal models, healthy volunteers and type 1 diabetic patients^62^. Emisphere Technologies are also pursuing oral delivery as such oral route of insulin delivery takes advantage of the portal-hepatic route of absorption, as insulin would be delivered to the liver, hence acting directly on hepatic glucose production in the same way of normal physiological state^63^.

### The patch:

Noninvasive transdermal insulin delivery could provide diabetic patients with sustained physiological levels of basal insulin in a pain-free manner^64^. Patches are the examples to deliver basal insulin rather than fast-acting boli in two steps process. First, by a device that would make microscopic holes in the top layer of the skin and secondly, the application of patch over the skin. Altea Development Corporation is planning to introduce a product which will either be a one- or half-day patch, depending on the outcome of testing.

## CONCLUSION

The ultimate goal in the treatment of type 1 diabetes mellitus would be probably some kind of β cells transplantation of the islets of Langerhans as a physiological alternative to insulin injections^65^,^66^or the correction of defective genes that are responsible for it. In type 2, emphasis should be given to prevent or delay the disease, which many studies related to urbanization, modernization and the sedentary life-style^67^. The current advocacy of intensive insulin therapy regimens involving multiple daily subcutaneous injections places a heavy burden of compliance on patients and has prompted interest in developing alternative. Therefore, investigations into various means to administer insulin in a safe and effective manner should continue.

Oral administration of insulin using products such as tablet or capsule would be the major technological breakthrough if approved for marketing. Such a goal necessitates intensive work and commitment from pharmaceutical manufacturing companies. Inhalation delivery system for insulin (Exubera^®^) is the first alternative method of insulin delivery that has recently been introduced to the market after FDA approval. The product would need time to gain acceptance from other health authorities, to provide long-term safety profile and gain popularity among health care professionals and patients. Exubera^®^ will also have to meet challenges by similar but compact devices since the size of the inhaler (25 cm) is considered to be cumbersome. Exubera^®^ is not to be used by smokers and also not recommended for people with asthma, bronchitis, or emphysema. Furthermore, the device is not approved for use by children less than 18 years of age. These limitations together with the use of only short-acting insulin with the device mean that the door is open for many challenges to this product.
